# B-cell lymphocyte kinase polymorphisms rs13277113, rs2736340, and rs4840568 and risk of autoimmune diseases: A meta-analysis

**DOI:** 10.1097/MD.0000000000007855

**Published:** 2017-09-08

**Authors:** Chang Zeng, Cheng Fang, Hong Weng, Xiaoqing Xu, Tianyang Wu, Wenhua Li

**Affiliations:** a Hubei Key Laboratory of Cell Homeostasis, College of Life Sciences, Wuhan University; b Center for Evidence-Based and Translational Medicine, Zhongnan Hospital of Wuhan University, Wuhan, China.

**Keywords:** autoimmune disease, B-cell lymphocyte kinase, BLK, meta-analysis, polymorphism

## Abstract

**Background:**

B-cell lymphocyte kinase (BLK) is an inhibitor of B cells that has an important influence on several autoimmune diseases, but there is a lack of comprehensive analysis of its association with autoimmune diseases. Hence, it is meaningful to conduct a comprehensive analysis.

**Methods:**

A systematic literature search was performed on the PubMed, ScienceDirect, and Web of Science databases up to June 30, 2016. The data were extracted and quality-assessed before conducting the meta-analysis. The odds ratios (ORs) and 95% confidence intervals (95% CIs) were assessed with the STATA version 12.0 software. Subgroup and sensitivity analysis were conducted to explore potential sources of heterogeneity.

**Results:**

Altogether, 33 studies with 68,874 cases and 90,684 controls, 24 studies with 31,095 cases and 39,077 controls for rs13277113, 21 studies with 26,388 cases and 40,635 controls for rs2736340, and 4 studies with 11,391 cases and 10,972 controls for rs4840568 were included in this meta-analysis. The results revealed that the BLK rs13277113 and rs2736340 polymorphisms increased the risk of autoimmune diseases in the total analysis (A vs G: OR = 1.33, 95% CI = 1.27–1.39, *P* < .01; T vs C: OR = 1.34, 95% CI = 1.27–1.41, *P* < .01), and rs4840568 was positively associated with systemic lupus erythematosus (SLE) (A vs G: OR = 1.32, 95% CI = 1.22–1.43, *P* = .01).

**Conclusion:**

This meta-analysis shows that the BLK (rs13277113, rs2736340, rs4840568) polymorphisms may be a risk factor for developing autoimmune diseases, especially for Asian populations and SLE.

## Abstract 2

Supplemental Digital Content is available in the text

## 1 Introduction

Autoimmune diseases are a clinical manifestation of a broken immune tolerance and intermittent inflammation, which leads to significant mortality and morbidity.^[1]^ Nearly 100 autoimmune diseases have been discovered, and autoimmune diseases can occur at any age. Epidemiological studies have shown that autoimmune diseases affect 3% to 5% of the general population.^[2]^ The prevalence is increased in woman, with the ratio of affected females to males ranging from 10:1 to 1:1.^[3–5]^ Generally, a broken immune tolerance is the key cause for autoimmune diseases. Immune tolerance is the ability to prevent the immune system from targeting self-molecules, cells, or tissues.^[6]^ When immune tolerance has been destroyed and self-reactive lymphocytes contribute to inflammation, a classical or pathological autoimmune disease develops. In this process, B cells, T cells, antigen-presenting cells, and effector cells all regulate the manifestation of autoimmune diseases.^[7–9]^

BLK is located on the short arm of chromosome 8 (8p23.1) and is one of the tyrosine kinases that transduce signals downstream of the B-cell receptor.^[10]^ A genome-wide association study (GWAS) confirmed that a single-nucleotide polymorphism (SNP) in the 5′ upstream region of the *BLK* gene was associated with SLE.^[11]^ Multiple studies found that BLK was also associated with other autoimmune diseases.^[12]^ Studies have shown that BLK is mainly expressed in B-cells and plays an important role in the proliferation and differentiation of B cells.^[13]^ Wu et al^[14]^ found that BLK risk genotypes in mice and humans were correlated with an increased subset of B1a or B1-like cells. Moreover, the expression of IgG anti-dsDNA antibodies in healthy individuals with a BLK risk genotype was significantly increased.^[14]^ Therefore, through regulating immunological components, the BLK risk genotypes might contribute to the development of autoimmune disease.

The A allele of rs13277113, T allele of rs2736340, and A allele of rs4840568 are located in the promoter region of BLK and inhibit the expression of BLK mRNA. Studies have shown that the BLK rs13277113 “A” allele was associated with an increased risk of a number of autoimmune diseases and other disorders, including SLE,^[15]^ systemic sclerosis (SSc),^[16]^ and rheumatoid arthritis (RA).^[17]^ Many randomized controlled trials (RCTs) have explored the relationship between BLK rs13277113 and other autoimmune diseases, such as polymyositis (PM),^[18]^ dermatomyositis (DM),^[18]^ multifocal motor neuropathy (MMN),^[19]^ and giant cell arteritis (GCA).^[20]^ Several studies have also shown that BLK rs2736340 was associated with SLE,^[21]^ and Kawasaki disease,^[22]^ and is possibly involved in RA,^[23]^ PM,^[24]^ DM,^[24]^ GCA,^[20]^ primary Sjögren syndrome (PSS),^[25]^ primary antiphospholipid syndrome (PAS),^[26]^ and SSc.^[27]^ Until now, studies found that BLK rs4840568 was merely related to SLE.^[28]^ In 2011, Fan et al^[15]^ performed a meta-analysis of 7 case–control studies for rs13277113 and found that the A allele of rs13277113 was a risk factor for SLE. Recently, another meta-analysis of 21 case–control studies for rs2736340 by Zhou et al,^[29]^ published in 2016, found that the rs2736340 polymorphism is associated with several autoimmune diseases. Small sample size and the low statistical power of previous studies contributed to our interest in conducting a comprehensive and precise analysis of the association between BLK polymorphisms and autoimmune diseases. This meta-analysis was conducted based on the meta-analysis of observational studies in epidemiology (MOOSE) guidelines.

## 2 Methods

Ethical approval was not unnecessary, as this study was a meta-analysis that collects and analyzes data from the existing literatures.

### 2.1 Eligibility criteria

The eligible studies met the following criteria were considered to include the study type is a case–control study or cohort study, investigated the association of the BLK (rs13277113, rs2736340, rs4840568) polymorphisms with autoimmune diseases susceptibility, included accurate genotype distribution data for each group or sufficient data to calculate the odds ratios (ORs) and corresponding 95% confidence intervals (CIs), and there were no limiting conditions regarding language. We excluded the abstracts, overlapped studies, and the study lack of key data.

### 2.2 Search strategy

The association between the BLK (rs13277113, rs2736340, rs4840568) polymorphisms and autoimmune diseases susceptibilities was explored by a comprehensive search. PubMed, ScienceDirect, and Web of Science were searched up to June 30, 2016, to identify the studies that used the following keywords: “BLK,” “B-cell lymphocyte kinase,” “autoimmune diseases,” “polymorphism,” “mutation,” “variant.” All of the included studies were published.

### 2.3 Data extraction

The following information was extracted independently by 2 authors (Zeng and Weng) with discussion: surname first author, publication year, country, the racial descent of the subjects, the source of the controls, the number of cases and controls with each genotype, Hardy–Weinberg equilibrium (HWE) among the controls, genotyping method, and type of diseases. The x^2^ test was used to estimate whether the genotype frequencies in the control groups accorded with HWE.

### 2.4 Quality assessment

Two independent authors (Zeng and Xu) evaluated the included case control studies using the Newcastle–Ottawa scale (NOS) for quality assessment. The scores on this scale range from 0 to 9 points. Studies with scores of 6 or higher were considered high quality; otherwise, they were identified as low quality.^[30]^

### 2.5 Statistical analysis

ORs with 95% CIs were calculated to assess the association between the BLK (rs13277113, rs2736340, rs4840568) polymorphisms and autoimmune diseases susceptibility. For the (rs13277113, rs2736340, rs4840568) polymorphisms, pooled ORs were used for the allele contrast (A vs G or T vs C), homozygote contrast (AA vs GG or TT vs CC), heterozygote contrast (AG vs GG or TC vs CC), the dominant model (AA+AG vs GG or TT+TC vs CC), and the recessive model (AA vs AG + GG or TT vs TC+CC). Subgroup analysis was based on the HWE status of control, ethnicity, the source of the controls, and types of diseases. The values of *P* < .1 and *I*^2^ > 50% were used to indicate the absence or existence of obvious heterogeneity; hence, a random-effect model was adopted. Otherwise, the fixed-effects model was used. Publication bias was analyzed with funnel plots. All of the statistical analyses were conducted using STATA version 12.0 (Stata Corporation, College Station, TX).

## 3 Results

### 3.1 Characteristics of included studies

**Figure 1:**
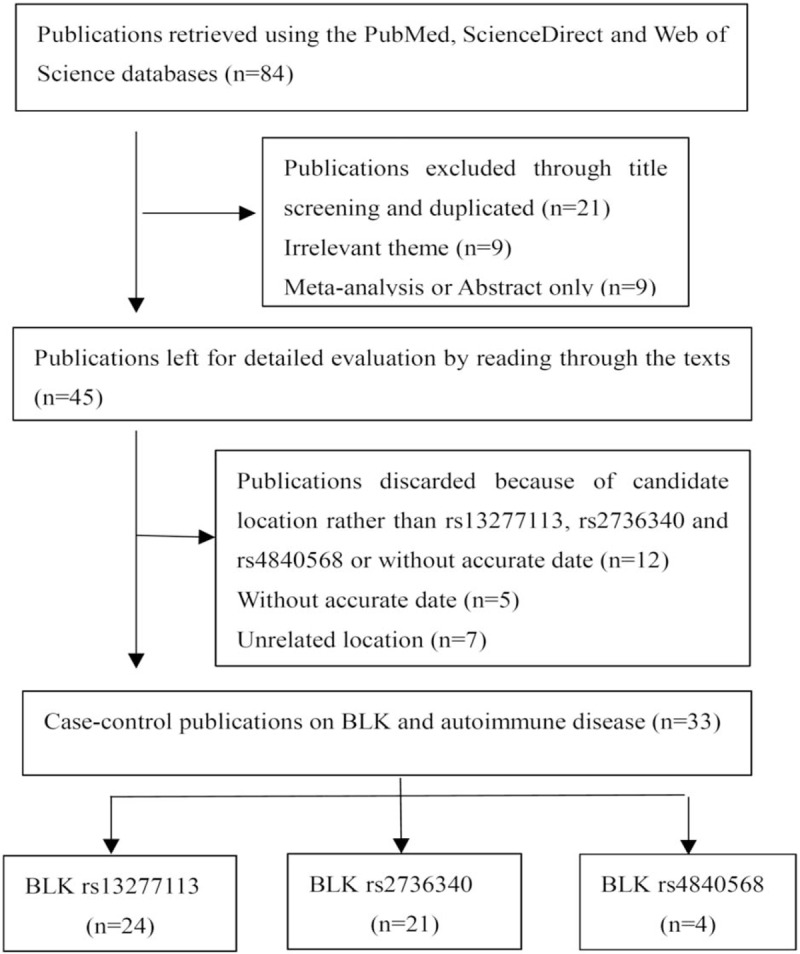
Flow diagram of the study selection process.

The initial literature search identified 84 relevant articles from the PubMed, ScienceDirect, and Web of Science databases. On the basis of title screening and duplications, 39 publications were excluded because of an irrelevant theme or were a duplicate study. Twelve publications were discarded because of irrelevant location or inaccurate data. This resulted in 33 significant articles for inclusion in our meta-analysis (Fig. 1).^[11,12,16,17,19,20,23–28,31–51]^ The specific characteristics and quality scores are provided in Table 1. All of the studies were case–control studies. Of these 33 articles, 4 articles reported on different countries,^[11,17,27,34]^ 2 articles reported on different ethnicities,^[31,42]^ 4 articles reported on different types of systemic scleroses or idiopathic inflammatory myopathies,^[24,27,35,44]^ and 13 articles reported on different sites of BLK polymorphisms^[11,20,23–25,27,28,33,36,38,41–43]^ and were treated as independent studies. Therefore, we divide the 33 articles into 66 specific case–control studies of BLK (68,874 cases and 90,684 controls).

**Table 1:**
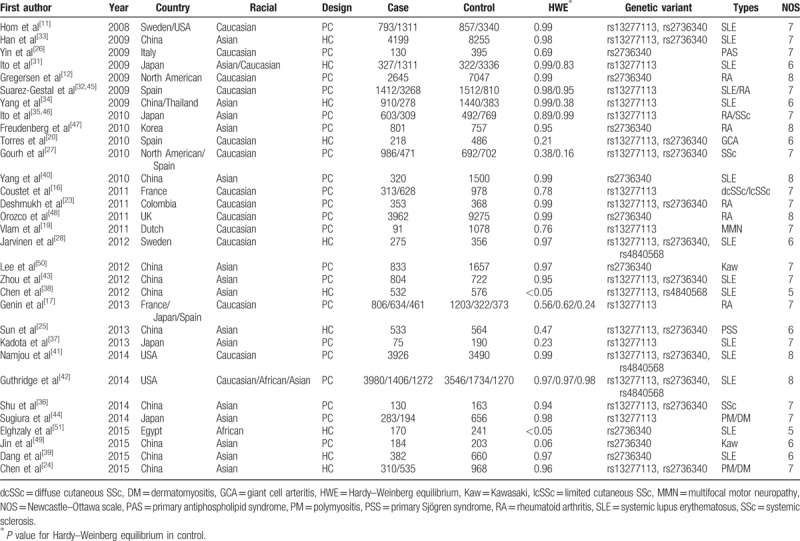
Characteristics of case–control studies on BLK (rs13277113, rs2736340, rs4840568) polymorphisms and autoimmunity disease risk included in the meta-analysis.

### 3.2 Meta-analysis

**Figure 2:**
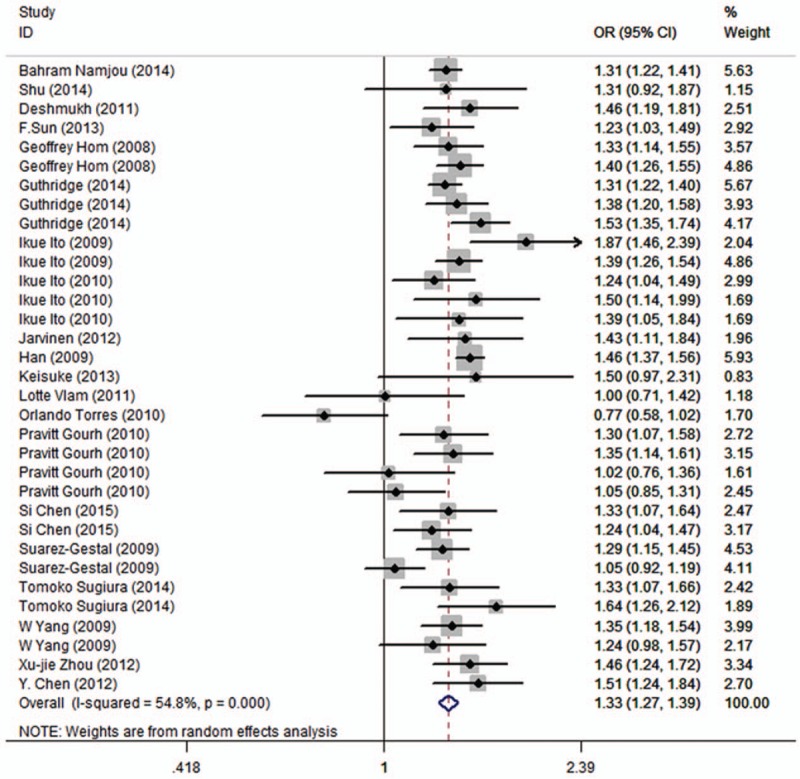
Calculated ORs and 95% CIs for the association between BLK rs13277113 polymorphism and autoimmune diseases risk in the A versus G model. 95% CIs = 95% confidence intervals, ORs = odds ratios.

The association between the rs13277113 polymorphism and autoimmune diseases risk is illustrated in Table 2. When these types of autoimmune diseases were viewed as a whole, we found that the combined analysis revealed a mildly increased risk of autoimmune diseases in 5 genetic models for BLK rs13277113 (A vs G: OR = 1.33, 95% CI 1.27–1.39, *P* < .01; AG vs GG: OR = 1.31, 95% CI 1.26–1.37, *P* = .6; AA vs GG: OR = 1.76, 95% CI 1.60–1.93, *P* < .01; AA+AG vs GG: OR = 1.41, 95% CI 1.33–1.51, *P* < .01; AA vs AG+GG: OR = 1.48, 95% CI 1.41–1.56, *P* = .16, Table 2, Fig. 2). The subgroup analysis by ethnicity indicated that the A allele was associated with an increased risk in Caucasians, Asians, and Africans (Table 2). It deserves to be mentioned that positive correlations with autoimmune diseases were observed in SLE (A vs G: OR = 1.39, 95% CI 1.34–1.44, *P* = .2). An association with dcSSc and lcSSc was apparent (A vs G: OR = 1.27, 95% CI 1.04–1.54, *P* = .16; A vs G: OR = 1.25, 95% CI 1.05–1.49, *P* = .15), as well as RA (A vs G: OR = 1.22, 95% CI 1.01–1.48, *P* = .02). The results of the subgroup analysis reveal that there are associations of the BLK rs13277113 polymorphism with PPS, PM, and DM but not with GCA (Table 3). However, due to small sample sizes, some of results were not statistically significant, such as MMN (Table S1). Therefore, the heterogeneity may have been derived from ethnicity, the source of the control, and the types of diseases and so on.

**Table 2:**
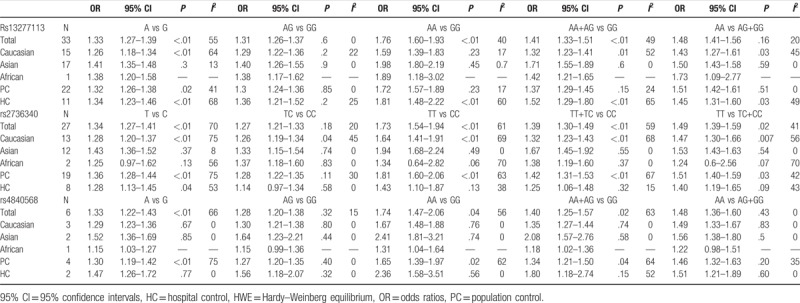
Summary ORs and 95% CI of BLK (rs13277113, rs2736340, rs4840568) polymorphisms and autoimmune disease risk by ethnicity and source of controls.

**Table 3:**
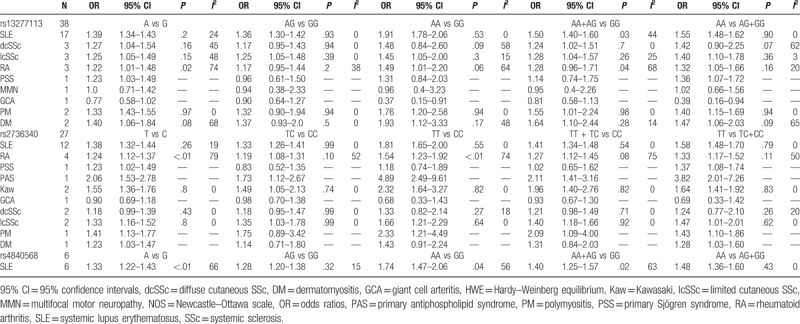
Meta-analysis for the association between BLK (rs13277113, rs2736340, rs4840568) polymorphism and autoimmune diseases by types of diseases.

**Figure 3:**
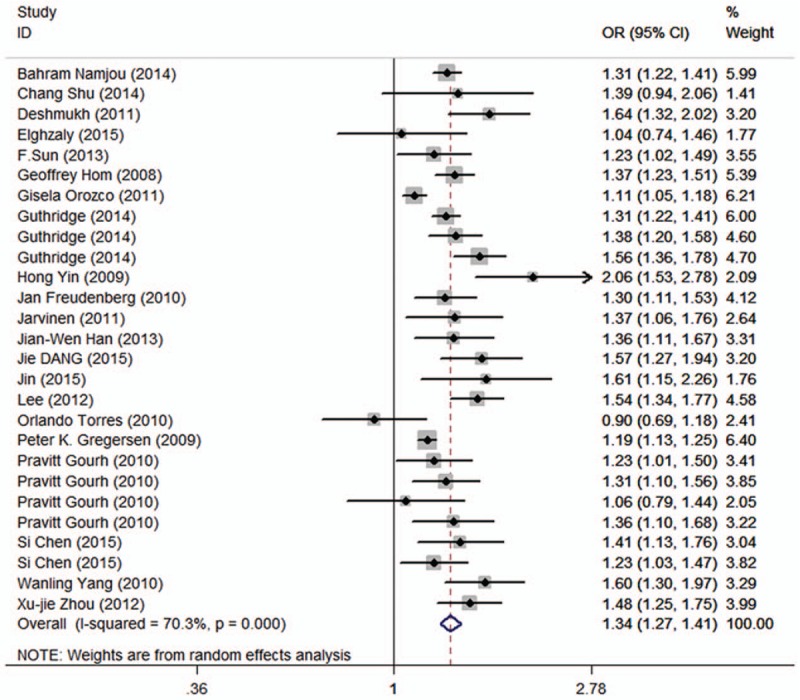
Calculated ORs and 95% CIs for the association between BLK rs2736340 polymorphism and autoimmune diseases risk in the T versus C model. 95% CIs = 95% confidence intervals, ORs = odds ratios.

For rs2736340, the pooled analysis merging all of the studies suggested a slight association with autoimmune diseases in 5 genetic models (T vs C: OR = 1.34, 95% CI = 1.27–1.41, *P* < .01; TC vs CC: OR = 1.27, 95% CI 1.21–1.33, *P* = .18; TT vs CC: OR = 1.73, 95% CI 1.54–1.94, *P* < .01; TT+TC vs CC: OR = 1.39, 95% CI 1.30–1.49, *P* < .01; TT vs TC+CC: OR = 1.49, 95% CI 1.39–1.59, *P* = .02, Table 2, Fig. 3). The subgroup analysis by ethnicity revealed that the T allele was a risk allele in Caucasians and Asians, but not in Africans. Positive correlations with autoimmune diseases were observed in SLE (OR = 1.38, 95% CI = 1.32–1.44, *P* = .26), RA (OR = 1.24, 95% CI = 1.12–1.37, *P* < .01), Kaw (OR = 1.55, 95% CI = 1.36–1.76, *P* = .8), and lcSSc (OR = 1.33, 95% CI = 1.16–1.52, *P* = .8) (Table 3). As before, the results of GCA and dcSSc were not statistically significant, larger sample sizes are needed (Table S1).

**Figure 4:**
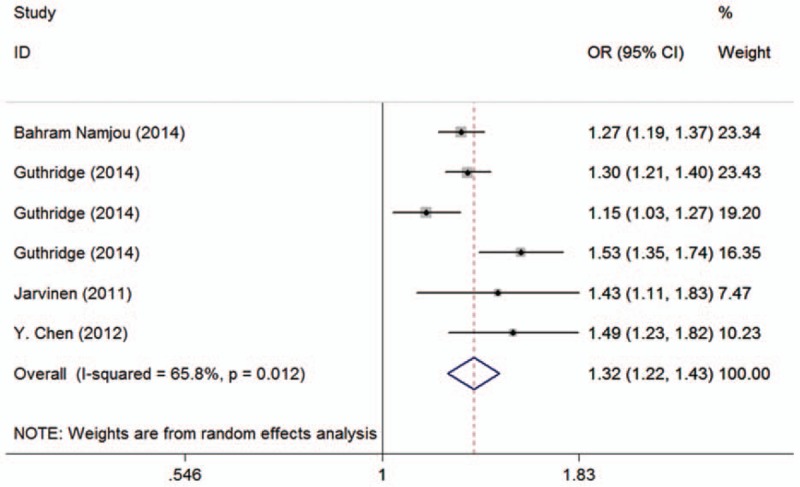
Calculated ORs and 95% CIs for the association between BLK rs4840568 polymorphism and autoimmune diseases risk in the A versus G model. 95% CIs = 95% confidence intervals, ORs = odds ratios.

As for rs4840568, we identified 6 studies, including 11,391 cases and 10,972 controls. Results revealed that rs4840568 polymorphism increased the risk of SLE in the total analysis (A vs G: OR = 1.32, 95% CI 1.22–1.43, *P* < .01; AG vs GG: OR = 1.28, 95% CI 1.20–1.38, *P* = .32; AA vs GG: OR = 1.74, 95% CI 1.47–2.06, *P* = .04; AA+AG vs GG: OR = 1.40, 95% CI 1.25–1.57, *P* = .02; AA vs AG+GG: OR = 1.48, 95% CI 1.36–1.60, *P* = .43, Table 2, Fig. 4).

### 3.3 Sensitivity analysis and Publication bias

**Figure 5:**
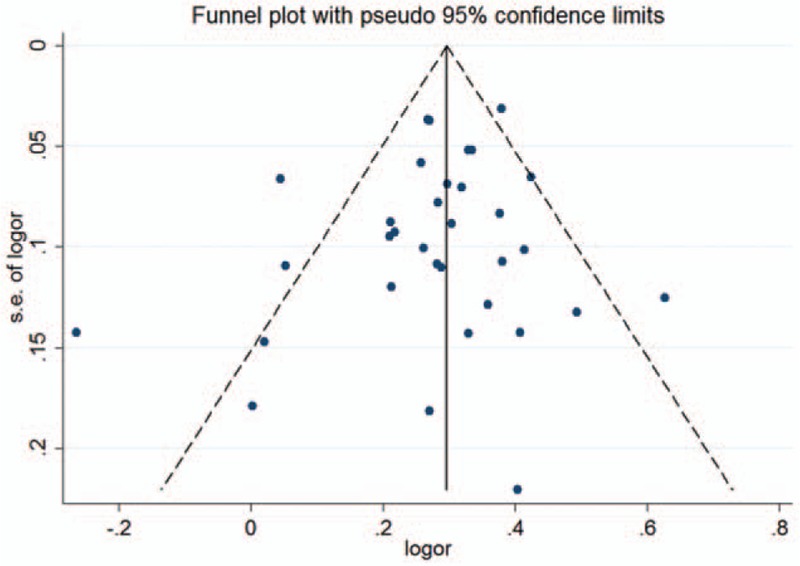
Funnel plot analysis to detect publication bias for A versus G model of BLK rs13277113 polymorphism circles represent the weight of the studies. ORs = odds ratios.

**Figure 6:**
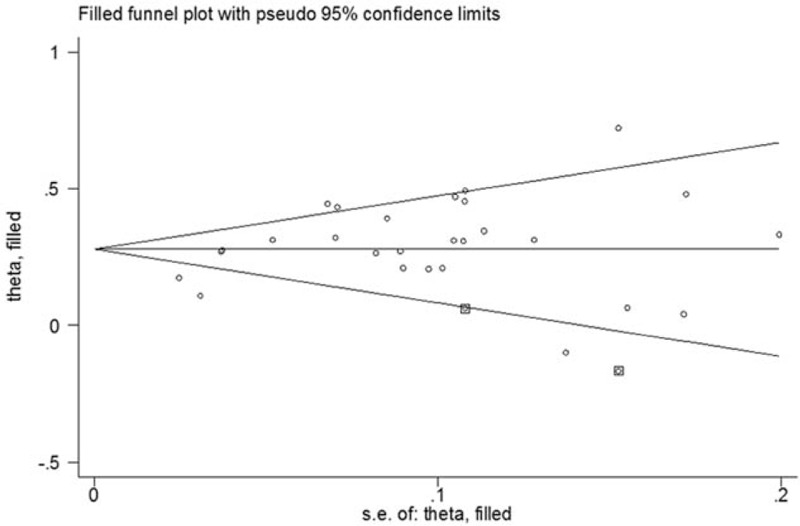
Funnel plot analysis with “trim and fill” method to expected publication bias for A versus G model of BLK rs2736340 polymorphism circles represent the weight of the studies. ORs = odds ratios.

In the sensitivity analysis, the pooled ORs were not substantially altered when any single study was excluded, which confirms that the results of this meta-analysis are statistically stable. For rs13277113 and rs4840568, the funnel plots displayed symmetrical shapes (Fig. 5). This result was supported by statistical evidence from Egger test (*P* = .269; *P* = .338), but for rs2736340, Egger test showed a publication bias (*P* = .013). The “trim and fill” method was used to mirror the asymmetric studies by imputing hypothetical negative unpublished studies. The pooled results with merged hypothetical studies still show an association with autoimmune diseases (OR = 1.32, 95% CI 1.25–1.39, *P* < .01), and the funnel plots using the “trim and fill” method displayed symmetrical shapes (Fig. 6).

## 4 Discussion

Genetic factors have become increasingly important in the progression of autoimmune diseases. In the pathogenesis and clinical manifestations of autoimmune disease, immune complexes in the bloodstream consisting of auto-antibody and antigen complexes lead to inflammation of the kidneys, brain, and skin.^[11]^ BLK is a member of the Src family of tyrosine kinases, which is involved in signal transduction downstream of the B-cell receptor (BCR).^[18]^ Previous studies have discovered the pathogenesis of mutant BLK in which the BCR triggers the phosphorylation of tyrosine residues on immunoreceptor via BLK and other Src family tyrosine kinases, inducing the phosphorylation and activation of tyrosine-protein kinase Syk. Syk is the key factor in initiating the pre-BCR signaling cascade, which leads to B cell activation and B cell–T cell interaction.^[23]^ In other words, the BLK risk genotypes lower the baseline for activation of B lymphocytes and assist in the communication between B lymphocytes and T lymphocytes. Thus, BLK has been deemed a meaningful candidate gene for autoimmune diseases.^[27,52]^

The findings from this meta-analysis, based on 68,874 cases and 90,684 controls of autoimmune diseases patients, found that the BLK polymorphisms rs13277113, rs2736340, and rs4840568 are significantly associated with susceptibility to autoimmune disease. The results were in accordance with previous studies and might provide a new biomarker in the etiology of autoimmune diseases. We also conducted subgroup analyses to further explore the potential effects of the patients’ ethnicities, the source of the controls, and the types of illness with the association of BLK polymorphisms and autoimmune diseases. The results revealed that BLK polymorphisms were closely associated with the risk of autoimmune diseases in all ethnicities; however, the correlation was stronger in Asian group than in other racial groups. Our studies also found that the BLK polymorphisms rs13277113, rs2736340, and rs4840568 are unevenly distributed in terms of ethnicity. In Caucasians and Africans, the A and T allele of BLK are the minor alleles, but they are the major alleles in Asians. Even so, the results indicated that the A allele of BLK rs13277113 and rs4840568 and the T allele of BLK rs2736340 were a risk factor for autoimmune diseases. The reason for this finding may be genetic disparities between the ethnic groups.^[15]^ Due to the process of natural selection, different groups might have some differences in the functional variants.^[51]^ In addition, the A allele of rs13277113 is a risk factor for SLE, diffuse cutaneous SSc (dcSSc), PSS, PM, and DM but is a protective factor for limited cutaneous SSc (lcSSc), RA, SSc, MMN, and GCA. As Zhou et al^[29]^ demonstrated, the T allele of rs2736340 is a risk factor in SLE, RA, Kawasaki disease, and lcSSc but was ambiguous with other diseases. Although autoimmune diseases have similar pathogenic mechanisms, there are some factors that cause differences in these diseases. This may be attributed to the fact that the majority of autoimmune diseases have multiple genetic factors that play a role.^[2]^ The number of studies included in this meta-analysis is an important aspect of these results. Our research found that BLK can act as an influencing factor on autoimmune diseases. Mutations of these SNPs can be used as a risk factor for assessing autoimmune diseases. Furthermore, on the basis of the pathogenicity of BLK polymorphisms, our research provides a meaningful therapeutic strategy for autoimmune diseases.

Recently, a similar meta-analysis by Zhou et al has been published online (https://www.ncbi.nlm.nih.gov/pubmed/27105348). We analyzed their well-designed study and found some advantages of our meta-analysis. In their study, they conducted a meta-analysis focusing on 21 studies and concluded that the BLK rs2736340 polymorphism is associated with several autoimmune diseases. We conducted a meta-analysis using 27 studies and obtained similar conclusions. However, the methodology assessment and sources of heterogeneity of the included studies in our analysis were more detailed than in the previous study (Tables 1 and 3). In addition, we also included 33 studies of BLK rs13277113 and 6 studies of BLK rs4840568 to systematically explore the relationship between BLK and autoimmune diseases. Therefore, a larger sample size and more SNP types are included in the present meta-analysis.

Nevertheless, some limitations should be acknowledged. First, the possible effects of gene–gene and gene–environmental interactions on the risks of autoimmune diseases were not estimated due to the limited information available in the original papers. Second, publication bias may exist because we only included published articles that were identified through online database searching. Third, high heterogeneity was observed in all 5 genetic models of 2 types of gene polymorphism. Subgroup analysis indicated that the heterogeneity might have been derived from the design of the controls, the ethnicities, and the types of diseases.

## 5 Conclusion

This meta-analysis indicated that the BLK (rs13277113, rs2736340, rs4840568) polymorphisms were associated with an increased risk of autoimmune diseases. In a word, because of the limitation in the included studies, more studies are essential for a more accurate conclusion.

## Supplementary Material

Supplemental Digital Content
