# Supplementary material for: B-cell lymphocyte kinase polymorphisms rs13277113, rs2736340, and rs4840568 and risk of autoimmune diseases: A meta-analysis
Source: Medicine (Baltimore). 2017 Sep 8;96(36):e7855. doi: 10.1097/MD.0000000000007855 (PMC6392982; doi:10.1097/MD.0000000000007855)
Supplement: Supplemental Digital Content [file medi-96-e7855-s001.pdf]

Supplementary Table S1

B-cell lymphocyte kinase polymorphisms rs13277113, rs2736340 and rs4840568 and the risk of autoimmune diseases: a meta-analysis

Chang Zeng, MD[a]; Cheng Fang, PhD[b]; Hong Weng, MDS[b]; Xiaoqing Xu, MD[a]; Tianyang Wu, MD[a]; Wenhua Li, PhD [a]*

Table S1. Statistical P values of BLK (rs13277113, rs2736340, rs4840568) polymorphisms by Ethnicity, source of controls and types of illness.

SNPs	N	P				
Rs13277113		AvsG	AGvsGG	AAvsGG	AA+AGvsGG	AAvsAG+GG
Total	33	<0.001	<0.001	<0.001	<0.001	<0.001
Caucasian	15	<0.001	<0.001	<0.001	<0.001	<0.001
Asian	17	<0.001	<0.001	<0.001	<0.001	<0.001
African	1	<0.001	<0.001	<0.001	<0.001	<0.001
PC	22	<0.001	<0.001	<0.001	<0.001	<0.001
HC	11	<0.001	<0.001	<0.001	<0.001	<0.001
SLE	17	<0.001	<0.001	<0.001	<0.001	<0.001
dcSSc	3	0.018	0.145	0.175	0.030	0.133
lcSSc	3	0.011	0.012	0.023	0.017	0.006
RA	3	0.042	0.132	0.044	0.091	0.017
PSS	1	0.026	0.842	0.236	0.556	0.012
MMN	1	0.99	0.893	0.933	0.914	0.946
GCA	1	0.064	0.543	0.031	0.218	0.036
PM	2	<0.001	0.162	0.004	0.020	0.001
DM	2	0.016	0.108	0.018	0.016	0.020
rs2736340		TvsC	TCvsCC	TTvsCC	TT+TCvsCC	TTvsTC+CC
Total	27	<0.001	<0.001	<0.001	<0.001	<0.001
Caucasian	13	<0.001	<0.001	<0.001	<0.001	<0.001
Asian	12	<0.001	<0.001	<0.001	<0.001	<0.001
African	2	<0.001	<0.001	<0.001	<0.001	<0.001
PC	19	<0.001	<0.001	<0.001	<0.001	<0.001
HC	8	<0.001	<0.001	<0.001	<0.001	<0.001
SLE	12	<0.001	<0.001	<0.001	<0.001	<0.001
RA	4	<0.001	<0.001	<0.001	<0.001	<0.001
PSS	1	0.034	0.457	0.496	0.929	0.009
PAS	1	<0.001	0.013	<0.001	<0.001	<0.001
Kaw	2	<0.001	0.027	<0.001	<0.001	<0.001
GCA	1	0.456	0.908	0.307	0.676	0.310
dcSSc	2	0.052	0.142	0.251	0.077	0.379
lcSSc	2	<0.001	0.001	0.002	<0.001	0.016
PM	1	0.002	0.104	0.012	0.026	0.008
DM	1	<0.001	0.592	0.121	0.234	0.024
rs4840568		AvsG	AGvsGG	AAvsGG	AA+AGvsGG	AAvsAG+GG
Total	6	<0.001	<0.001	<0.001	<0.001	<0.001
Caucasian	3	<0.001	<0.001	<0.001	<0.001	<0.001
Asian	2	<0.001	0.001	<0.001	<0.001	<0.001
African	1	0.011	0.072	0.021	0.023	0.071
PC	4	<0.001	<0.001	<0.001	<0.001	<0.001
HC	2	<0.001	0.002	<0.001	0.007	<0.001
SLE	6	<0.001	<0.001	<0.001	<0.001	<0.001
